# Circuit Mechanisms of Memory Formation

**DOI:** 10.1155/2011/494675

**Published:** 2011-12-07

**Authors:** Björn M. Kampa, Anja Gundlfinger, Johannes J. Letzkus, Christian Leibold

**Affiliations:** ^1^Department of Neurophysiology, Brain Research Institute, University of Zurich, 8057 Zurich, Switzerland; ^2^Friedrich Miescher Institute for Biomedical Research, Maulbeerstraße 66, 4058 Basel, Switzerland; ^3^Department of Biology II, University of Munich, 82152 Planegg-Martinsried, Germany

## 1. Introduction

Memory formation is one of the most fascinating and complex brain functions. A large body of research over the last decades has drastically increased our understanding of the molecular and cellular processes underlying learning, most notably through a detailed investigation of synaptic plasticity. This reductionist approach, typically involving *in vitro* experiments, has been tremendously successful in providing a mechanistic framework for learning at the level of single neurons. However, real-life memories are formed through dynamic interactions of many neurons embedded in large networks. Investigating the mechanisms and consequences of learning at the level of neuronal circuits is technically much more demanding, and we are only beginning to understand this important topic. This special issue presents recent progress in illuminating the most exciting issues in the field of *circuit mechanisms of memory formation*. The contributing articles cover essential concepts and hypotheses underlying memory formation ranging from synaptic mechanisms of plasticity in neuronal microcircuits to circuit reorganizations in response to physiological and pathological influences. 

## 2. Plasticity of Inhibitory Circuits

Neuronal circuits are assembled by intricately interconnected excitatory and inhibitory units. The balance of excitation and inhibition is absolutely critical to circuit function. The lion's share of research into plasticity has focused on excitatory synapses. However, GABAergic inhibition in the cortex plays a major role in development and ocular dominance plasticity as reviewed by Heimel et al. Sensory deprivation of the visual input through one eye leads to the dominance of the contralateral eye's inputs. This also changes the ocular preference of cortical neurons during the sensitive period. One of the central mechanisms responsible for opening the sensitive period is the maturation of inhibitory innervation, which may also involve plasticity of inhibitory inputs. The many forms and functions of long-term plasticity at GABAergic synapses are reviewed by A. Maffei. New experimental work has demonstrated that inhibitory synapses also undergo plastic changes and follow their own learning rules. Understanding these rules is crucial to fully comprehend the circuit mechanisms of memory formation.

## 3. Neuromodulation of Learning and Memory

The effect of long-range neuromodulatory inputs on local circuit computations is at present a very dynamic field of investigation, in particular since neuromodulation is well known to be critical for many forms of learning. The cholinergic system is implicated in gating cortical plasticity during associative learning and sensory map plasticity. M. B. Verhoog and H. D. Mansvelder review how cholinergic modulation acting via presynaptic ionotropic receptors may create brief time windows for synaptic modulation during spike-timing-dependent plasticity. C. Köhler et al. address the modulation of hippocampal and neocortical memory systems by the relatively little known neuromodulator histamine. They provide an overview of the anatomy of histaminergic systems, histamine metabolism, receptors, and turnover and introduce the involvement of histamine in synaptic plasticity. Finally, K. Okada et al. discuss how a prominent neuromodulatory signal during learning, the dopaminergic prediction error signal, is computed with a special emphasis on the involvement of other neuromodulatory systems.

## 4. Motor Learning

Circuit plasticity in sensorimotor areas has become a major interest since the recent introduction of brain-machine interfaces. Focusing on motor learning in the rat, J. Francis and W. Song discuss plasticity mechanisms on the behavioral, neurophysiological, and synaptic levels. In addition, the authors present data on the inhibition of protein kinase M*ζ*, which is necessary for the maintenance of long-term potentiation. Relating molecular plasticity with behavioral changes, these results shed new light on circuit mechanisms of motor learning. These cortical circuits are organized by specific connections between their neuronal members. While many motifs of synaptic connectivity have been elucidated *in vitro*, we still know very little about the organization of network activity in the intact brain during behavior. D. F. Putrino et al. address this issue in cats, which were trained to perform skilled movement task. They describe dynamic spiking associations between single neurons in primary motor cortex, which were highly sensitive to spontaneous errors in task performance. J. A. Hosp and A. R. Luft finally review different physiological and pathological scenarios inducing neuroplasticity and circuit reorganization in the motor cortex. They differentiate between learning of novel movement sequences in healthy individuals, spontaneous cortical reorganization after ischemic injury, and relearning of skilled movement sequences under neurorehabilitative training in the injured brain. Although their main focus lies on studies in rodents and nonhuman primates, the authors also provide a useful outlook on the implications of the findings for clinical practice.

## 5. Circuit Mechanisms in Hippocampus and Amygdala

The hippocampal-entorhinal circuitry provides the most crucial anatomical and functional substrate for both memory formation and spatial navigation. An appealing network model for the consolidation of newly formed memory involves repetition of previously experienced spiking activity in the hippocampus during sleep. L. Buhry et al. contribute a comprehensive review over a large collection of papers on such hippocampal “replay” phenomena, which are correlated to neural sequences recorded during preceding exploration phases. They specifically focus on the relation of these activity sequences to memory consolidation during sleep. The point is made that all the different flavors of those sequence-like activities no longer allow to see those sequences as a mere memory replay phenomenon. The authors therefore propose the broader conceptual framework of offline sequential activity and discuss its possible relation to cognitive functions. In similar lines, J. H. L. P. Sadowski et al. review *in vivo* studies of hippocampal sharp wave ripple complexes and their relation to sequence replay phenomena. In contrast to the review by L. Buhry et al., they mostly focus on the cellular and circuit mechanism that could underlie this phenomenon. A particular emphasis is laid on how activity patterns can be transmitted between the different hippocampal and extrahippocampal regions during sharp wave oscillations and how synaptic plasticity may benefit from this dynamical state. K. J. Jeffery then introduces the reader to the general concepts and use of discrete and continuous attractor networks involved in spatial navigation. She reviews the evidence for the existence of such attractor networks and finally presents a hypothesis—on modeling and experimental work—on how partial remapping and attractor dynamics can coexist in the hippocampal-entorhinal network. Similar attractor models can explain the outcomes of a “teleportation experiment” described by F. Stella and A. Treves. In such an experiment, a rat was rapidly “teleported” from one familiar environment to another by changing the luminance conditions. The resulting hippocampal activity pattern alternated between the two representations for a short while, mostly without showing intermediate patterns, which indicates attractor-type dynamics. While the hippocampal formation and connected entorhinal cortex are the most studied systems for classical declarative memory formation, other types of learning are linked to deep-brain structures. Fear conditioning, for instance, is a powerful paradigm to investigate the neuronal mechanisms underlying associative learning. The amygdala is a critical brain area for this type of learning, and recent evidence indicates that it functions as a hub which integrates and orchestrates activity in a distributed network of brain areas during aversive learning. In this issue, H. Toyoda et al. review the involvement of the anterior cingulated cortex (ACC) in fear learning and compare the mechanisms of synaptic plasticity induction in ACC to those found in the amygdala.

In conclusion, this special issue summarizes a broad range of topics in the field of circuit plasticity and offers new insights and perspectives on linking molecular mechanisms with behavioral outcome of learning and memory. The presented work covers experimental data and also theoretical models that together can explain some of the intriguing phenomena of computation and memory formation in the brain.

*Björn M. Kampa* *Björn M. Kampa* *Anja Gundlfinger* *Anja Gundlfinger* *Johannes J. Letzkus* *Johannes J. Letzkus* *Christian Leibold* *Christian Leibold*

